# Disparities in Stillbirths in England: Analysis of A Population‐Based Study of 1.3 Million Births

**DOI:** 10.1111/1471-0528.18147

**Published:** 2025-05-16

**Authors:** Ggenga Kayode, Baskaran Thilaganathan, Christy Burden, Amy Howell, Vincent Cheng, Jane Sandall, Maria Viner, Lia Brigante, Dilly Anumba, Cathy Winter, Birte Harlev‐Lam, Timothy Draycott, Andrew Judge, Erik Lenguerrand, Tommy's National Centre for Maternity Improvement

**Affiliations:** ^1^ Translational Health Science, Bristol Medical School, Southmead Hospital University of Bristol Bristol UK; ^2^ Royal College of Obstetricians and Gynaecologists London UK; ^3^ St. George's University Hospitals London UK; ^4^ Department of Women and Children's Health, Faculty of Life Sciences & Medicine King's College London London UK; ^5^ Mothers for Mothers Bristol UK; ^6^ Royal College of Midwives London UK; ^7^ Academic Unit of Reproductive and Developmental Medicine‐Obstetrics and Gynaecology, Faculty of Medicine Dentistry and Health The University of Sheffield Sheffield UK; ^8^ The PROMPT Maternity Foundation, Department of Women's Health Southmead Hospital Bristol UK

**Keywords:** ethnicity, maternity care provision, social inequality, stillbirth

## Abstract

**Objective:**

To examine the variation in stillbirth rates between different ethnic and socioeconomic groups within each organisational hospital group (health trust).

**Design:**

National registry study.

**Setting:**

All health trusts (HT) in National Health Service England.

**Population:**

All mothers and babies born between April 2015 and March 2017.

**Methods:**

This observational study examined ethnic and socioeconomic disparities in stillbirth rates for 1 268 367 births in 133 HTs compared to the national average.

**Outcome:**

Stillbirth at or after 24 gestational weeks.

**Results:**

The average stillbirth rates ranged from 3.4/1000 births for White women up to 7.1/1000 births for Black women. The rates ranged from 2.9/1000 births for women living in the least deprived areas to 4.7/1000 births for those in the most deprived. The proportions of HTs with stillbirth rates well above the national average (more than 2 standard deviations) for White, Asian and Black women were 0.8%, 21.8% and 38.6%, respectively. When HTs were ranked by stillbirth rate, there were notable variations, with some trusts demonstrating lower than average stillbirth rates for White women while concurrently having higher than average stillbirth rates for Asian and/or Black women. There were no units exhibiting lower than national average stillbirth rates for Asian/Black women while concurrently having higher than average stillbirth rates for White women.

**Conclusions:**

These findings suggest that access to and delivery of maternity care vary depending on the mother's ethnicity and level of socioeconomic deprivation. Social factors are likely determinants of inequality in stillbirth rather than maternity care alone.

**Abbreviations**
IMD: index of multiple deprivation
LSOA: lower layer super output area
MIS: maternity information systems
NHS: National Health Service
NMPA: National Maternity and Perinatal Audit
SD: standard deviation
UK: United Kingdom

## 1 Introduction

Stillbirth has a devastating as well as protracted psychosocial and economic impact on families and society at large, making prevention of stillbirth a major global ambition [1, 2, 3]. Ethnicity, migration and socio‐economic status of women are strong determinants of adverse pregnancy outcomes and drivers of health inequity [4, 5, 6, 7, 8, 9, 10]. Despite this, there is a paucity of published data on disparities in stillbirth rates across health trusts based on maternal ethnicity and socio‐economic status.

Recent, national surveillance datasets within the United Kingdom (UK) show substantial variation in stillbirth rates, with stillbirth rates twice as high in Black women compared to White women, as well as variation in maternity units across the country [9, 11, 12, 13]. However, research into ethnic inequalities in stillbirth is limited in the UK. Current reports and published studies have focused mainly on descriptive analysis of geographical disparities in stillbirth and perinatal mortality without investigating the relationship to markers of health inequity and the possible impact of access to perinatal care [14, 15]. In particular, the extent of institutional bias on the associations between ethnicity and stillbirth has been suggested, but not systematically evaluated.

The aim of this study is to examine variation in stillbirth rates across different ethnic groups within organisational hospital groups (health trusts) and estimate the extent to which maternal ethnicity and socio‐economic status influence disparities. Such findings may provide vital information to help stakeholders offer more tailored and targeted services to reduce the impact of health inequity on stillbirth rates.

## 2 Methods

### 2.1 Study Population

This observational study used data from mothers and babies born in England from April 1, 2015, to March 31, 2017, captured on hospital maternity information systems (MIS)—a subset of the National Maternity and Perinatal Audit (NMPA) [16] data related to National Health Service (NHS) England maternity units with approval from the Healthcare Quality Improvement Partnership (DARS‐NIC‐430380‐F7L4Z‐v0.4 HQIP348). During the study period, 234 maternity units from 133 health trusts submitted specific maternity information in England. The MIS datasets cover about 97% of all total births in England, and the data are of high quality [17, 18]. Publicly available information describing the lower layer super output area (LSOA) were linked to the anonymised MIS dataset to provide information on the index of multiple deprivation (IMD) of maternal residential areas [19].

### 2.2 Outcome and Exposure

Stillbirth was defined as a baby born at or after 24 weeks gestational age with no sign of life. Maternal ethnicity was reported as recorded by healthcare providers: The NHS uses a standardised list of 16 categories to determine a patient's ethnicity. The NHS recommends that organisations ask patients about their ethnicity using self‐reporting, along with questions about national identity and religion. We classified ethnic groups as Asian, Black, mixed ethnicity, other and White [20, 21].

Women included in this analysis were categorised into 5 IMD groups; an aggregated index of socio‐economic deprivation of the maternal residential area was used as a proxy for socioeconomic status [19]. In England, deprivation is measured in small geographical areas known as LSOA. LSOAs are defined as geographical areas of a similar population size, with an average of 1500 residents. As a measure of socioeconomic deprivation, we used the IMD score, a relative measure of deprivation based on LSOAs. Publicly available information describing the LSOA, produced by the Office of National Statistics, was linked to the anonymised MIS dataset to provide information on the IMD of maternal residential areas [19]. The IMD is the most used measure of deprivation within small areas in England. The seven domains used to generate deprivation scores include income, employment, education, health, crime, barriers to housing and services, and living environment. We categorised IMD into five groups (quintiles), with 1 being the most deprived and 5 denoting the least deprived group.

### 2.3 Statistical Analysis

Participants' characteristics were reported as frequencies and percentages (%). Disparities in stillbirth rates were calculated for both individual maternity units and their amalgamated health trusts. Standard deviations [SD] were used to visualise stillbirth rate variation between health trusts as they are commonly in national audits [22]. The national average of stillbirth and the SD were determined across all Trusts included in the analysis. Using the national average of stillbirth and corresponding SD, Trusts were classified into five categories based on their stillbirth rates (Figure S1) Well below average (< −2 SD below the national average, Green), below average (−2 SD to −1 SD, Dark blue), average (−1 SD to +1 SD, Sky blue), above average (+1 SD to +2 SD, Orange) and well above average (> +2 SD, Red). Stillbirth rates estimated by maternal ethnicity and IMD were compared to the overall national average or national average by ethnic/socioeconomic group. We determined the average rate of stillbirth per each IMD and ethnic group, as well as the national stillbirth rate across all ethnic and IMD groups. Average stillbirth rates estimated by maternal ethnicity and IMD were compared to the national average. We then performed a one‐sample *t*‐test to compare the average stillbirth rate for each group to the national average. All statistical analyses were performed in RStudio statistical software package version 4.0.2 [23].

We determined the mean stillbirth rate in each health trust, with both the 95% and 99.8% confidence intervals around the mean for each individual health trust. We then plotted the national average of stillbirths to establish if the confidence intervals of each individual health trust are above or below the national average [24].

## 3 Results

**FIGURE 1:**
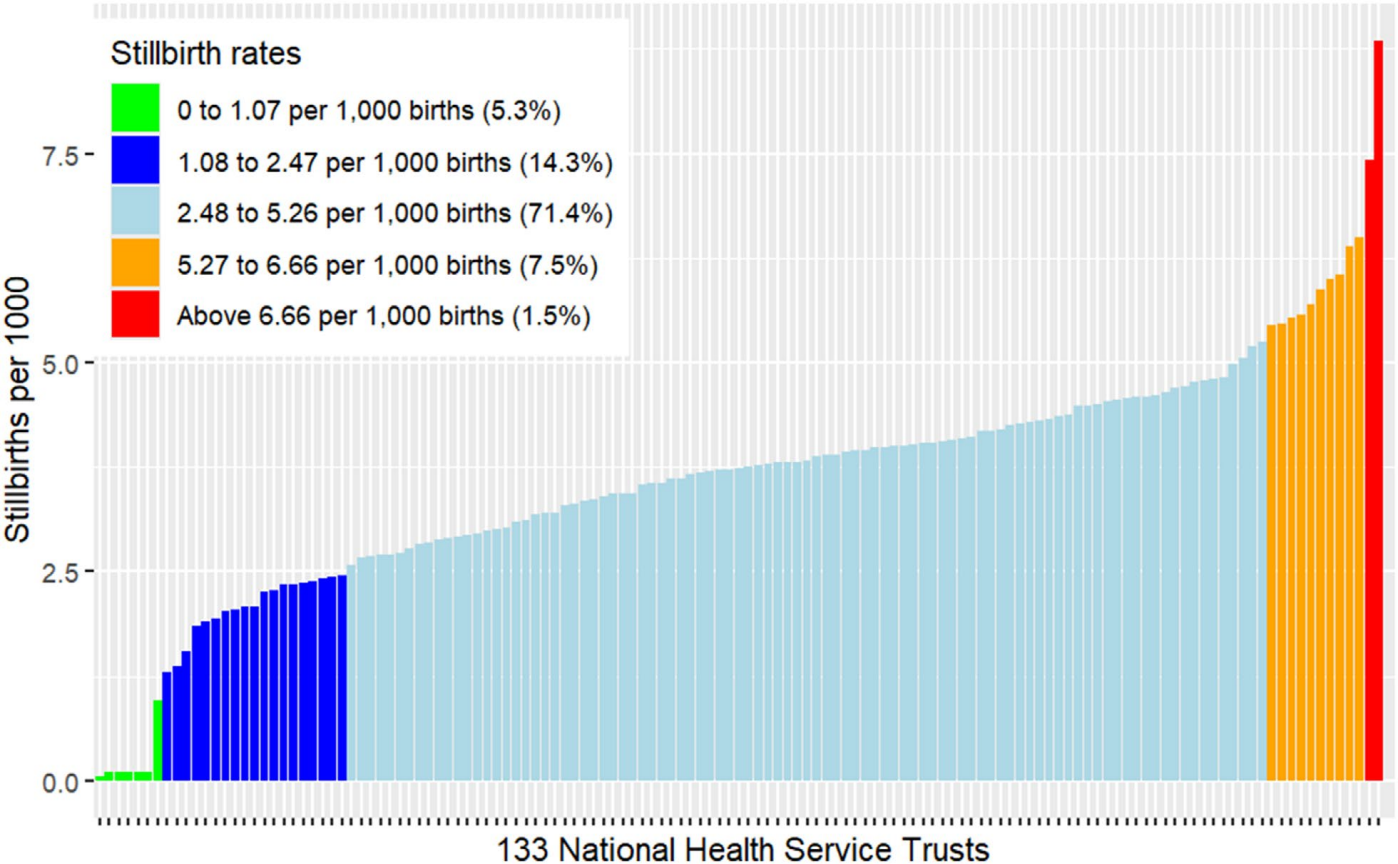
Variation in stillbirth rate across health trusts in NHS England compared to the national rate (3.4 stillbirths/1000 births).

The maternal characteristics for the 1 260 567 births (Figure S2) and 4890 stillbirths (3.4 stillbirths/1000 births) are shown in Table 1 Most stillbirths occurred in nulliparous women (41.1%, *n* = 1808), those aged 30–34 years (28.4%, *n* = 1383) and with a BMI between 18.5 and 25 kg/m^2^ (41.4%, *n* = 1503). However, the highest rates of stillbirth (4.4/1000 births) were observed at the extremes of maternal age (< 20 years and ≥ 35 years), respectively, in women with five or more births (7.5/1000 births) and those with a BMI ≥ 35 kg/m^2^ (5.3/1000 births). The variation in stillbirth rate across health trusts in NHS England is shown in Figure 1 and Figure S3. The proportion of health trusts classified as having stillbirth rates well below average was 5.3% (Green, *n* = 7), whereas the proportion classified as well above average was 1.5% (Red, *n* = 2).

**TABLE 1:** Participant characteristics.

Characteristics	Overall	Livebirths	Stillbirths	Stillbirth rate
	(*n* = 1 260 567)	(*n* = 1 255 677)	(*n* = 4890)	(Per 1000 births)
Maternal age (years)				
< 20	39 515 (3.2%)	39 340 (3.2%)	175 (3.6%)	4.4
20–24	184 745 (14.8%)	183 966 (14.8%)	779 (16.0%)	4.2
25–29	351 010 (28.1%)	349 706 (28.1%)	1304 (26.8%)	3.7
30–34	394 907 (31.6%)	393 524 (31.6%)	1383 (28.4%)	3.5
≥ 35	278 879 (22.3%)	277 652 (22.3%)	1227 (25.2%)	4.4
Unknown	11 511	11 489	22	
Parity				
0 (Nulliparous)	457 517 (40.6%)	455 709 (40.6%)	1808 (41.1%)	4.0
1	395 590 (35.2%)	395 305 (35.2%)	1285 (29.2%)	3.2
2	164 371 (14.6%)	163 689 (14.6%)	682 (15.5%)	4.1
3	64 262 (5.7%)	63 930 (5.7%)	332 (7.5%)	5.2
4	24 754 (2.2%)	24 607 (2.2%)	151 (3.4%)	6.1
≥ 5 (Grand multiparous)	19 127 (1.7%)	18 984 (1.7%)	143 (3.3%)	7.5
Unknown	133 942	133 453	489	
Body mass index (kg/m^2^)				
< 18.5	28 769 (2.9%)	28 667 (2.9%)	102 (2.8%)	3.5
18.5 to < 25	472 278 (48.2%)	470 775 (48.3%)	1503 (41.4%)	3.2
25 to < 30	272 786 (27.9%)	271 721 (27.9%)	1075 (29.6%)	3.9
30 to < 35	126 463 (12.9%)	125 931 (12.9%)	532 (14.7%)	4.2
≥ 35	78 685 (8.1%)	78 269 (8.0%)	416 (11.5%)	5.3
Unknown	281 576	280 314	1262	
Ethnicity				
Asian	136 383 (11.9%)	135 644 (11.9%)	741 (16.7%)	5.4
Black	57 774 (5.1%)	57 365 (5.1%)	409 (9.2%)	7.1
Mixed	21 734 (1.9%)	21 637 (1.9%)	97 (2.2%)	3.9
Others	48 994 (4.3%)	48 802 (4.3%)	192 (4.3%)	4.5
White	875 217 (76.8%)	872 217 (76.8%)	3000 (67.6%)	3.4
Unknown	120 463	120 012	451	
Index multiple deprivation				
1 (most deprived)	317 294 (26.7%)	315 799 (26.8%)	1495 (33.5%)	4.7
2	267 810 (22.6%)	266 747 (22.6%)	1063 (23.8%)	4.0
3	222 781 (18.9%)	221 984 (18.8%)	797 (17.9%)	3.6
4	198 009 (16.8%)	197 414 (16.8%)	595 (13.4%)	3.0
5 (least deprived)	177 334 (15.0%)	176 826 (15.0%)	508 (11.4%)	2.9
Unknown	77 339	76 907	432	

### 3.1 Stillbirth Rate Variation

**FIGURE 2:**
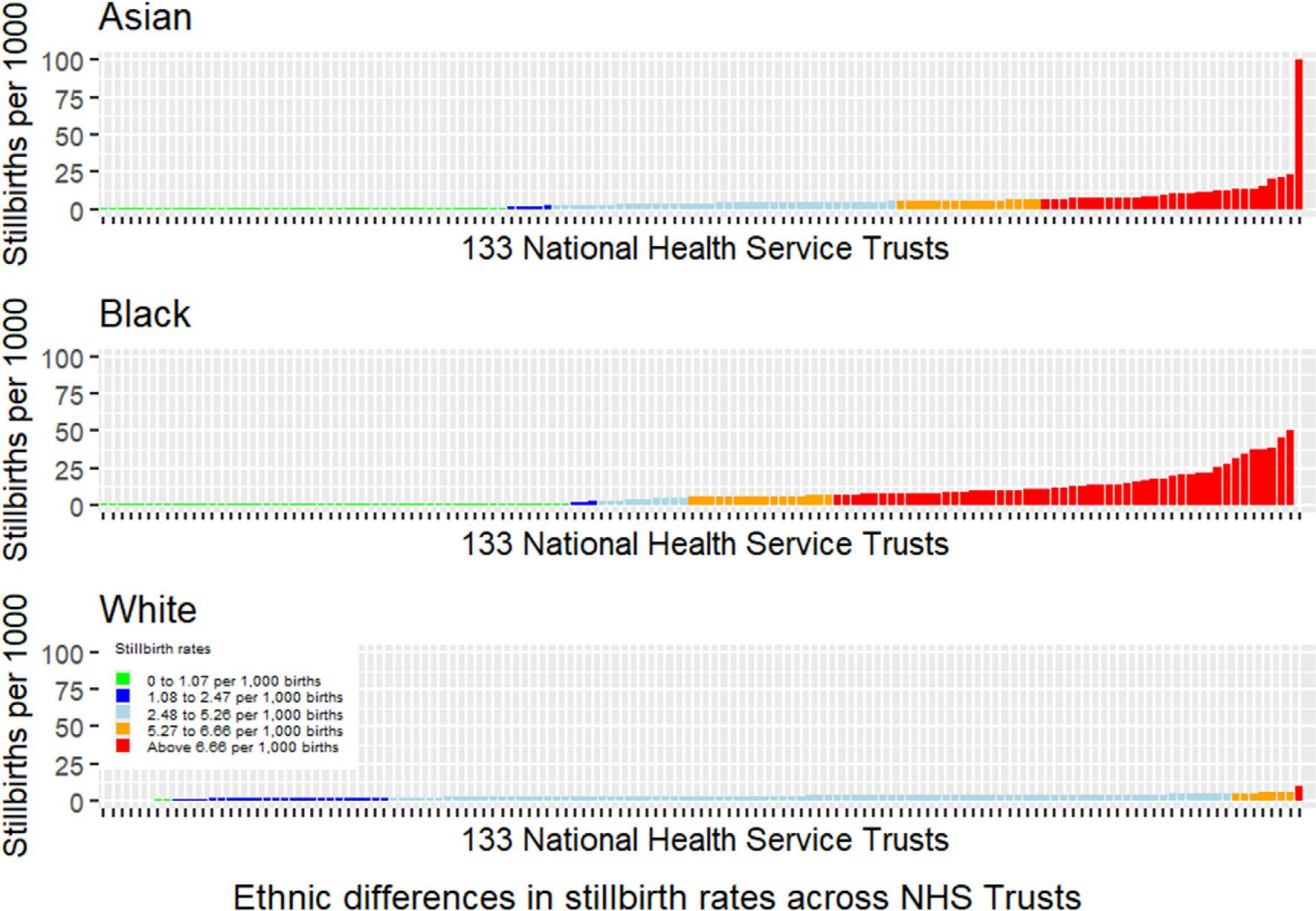
Variation in stillbirth rate across health trusts in NHS England by ethnicity compared to the national rate (3.4 stillbirths/1000 births).

The stillbirth rate in average health trusts (blue) was similar for all ethnicities, ranging from 3.7/1000 births in White women up to 4.1/1000 births for Asian women (Table 1). The stillbirth rates in the well above average (red) health trusts varied from 9.8/1000 births in White women to 15.6/1000 births in Black women. The proportions of health trusts with well above average (red) stillbirth rates for White, Asian and Black women were 0.8%, 21.8%, and 38.6%, respectively (Figure 2). When health trusts were ranked in the same order according to the overall stillbirth rate in White women, there were notable variations in rates of stillbirth within the same health trust for White, Asian and Black women (Figure S4). Some health trusts demonstrating lower than average stillbirth rates for White women concurrently demonstrated higher than average stillbirth rates for Asian and/or Black women. There were no units exhibiting lower than average stillbirth rates for Asian/Black women while concurrently having higher than average stillbirth rates for White women.

**FIGURE 3:**
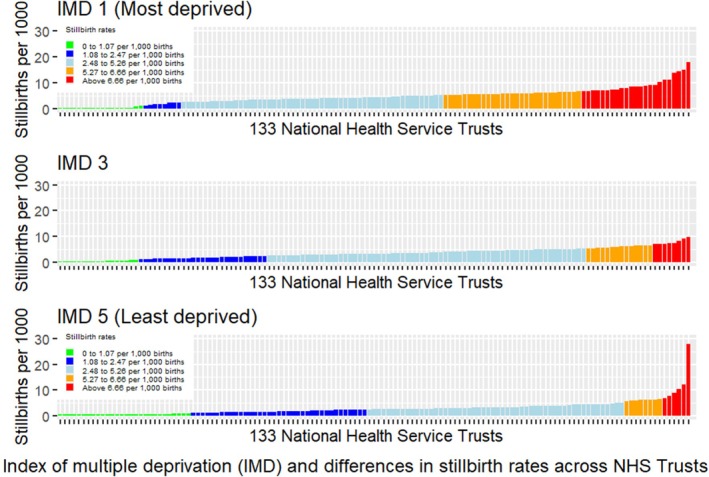
Variation in stillbirth rate across health trusts in NHS England by Index of Multiple Deprivation (IMD) compared to the national rate (3.4 stillbirths/1000 births).

The proportion of health trusts with well above average stillbirth rates was 4.5% for women living in the least deprived areas, while the corresponding figure was 17.3% for those living in the most deprived areas, respectively (Figure 3). When health trusts were ranked according to overall stillbirth rate in White women, there were notable variations in rates of stillbirth within the same health trust for the least and most deprived women (Figures S5 and S6). The proportions of health trusts with well above average stillbirth rates for White, Asian and Black women living in the most deprived areas were 15%, 27.8%, and 31.2%, respectively; The stillbirth rates in White, Asian and Black women from the most deprived areas were 4.3/1000 births, 6.7/1000 births and 5.7/1000 births, respectively (Figure S7). The rates from the least deprived areas were 2.6/1000 births, 5.9/1000 births, and 4.6/1000 births, respectively, with fewer trusts well above average stillbirth rates for White women (Figure S8). The rates of stillbirth by ethnicity or socioeconomic level are presented in Table S1.

## 4 Discussion

This study investigated stillbirths across 133 NHS health trusts in England and confirmed the health disparity conferred by both ethnicity and socioeconomic deprivation. Most notably, some health trusts reported below average stillbirth rates for White women and concurrently reported above average stillbirth rates for Black and Asian women delivering in the same trust. Similar findings were evident for women living in areas with the highest and lowest socioeconomic deprivation. Stillbirth rates are concordant with socioeconomic deprivation, with women living in the poorest areas having the highest stillbirth rates and accounting for the majority (IMD 1 and 2, *n* = 2802, 57.3%) of all stillbirths. Similarly, Black and Asian women had the highest stillbirth rates, accounting for 25.9% (*n* = 1150) of stillbirths.

A substantial portion of the variation in stillbirth rates occurring in England can be attributed to socioeconomic and ethnic inequality [10]. Ethnicity is a social construct with strong intersectionality with socioeconomic deprivation [25]. Our findings are consistent with previous studies conducted in the UK, Europe and North America, showing that ethnic minority mothers residing in the most socioeconomically deprived neighbourhoods were more likely to have stillbirths [4, 6, 9]. These could be attributed to the inverse relationship between socioeconomic deprivation and access to quality perinatal care [26, 27]. Considering the wide disparity in stillbirths among mothers of the same ethnicity or neighbourhood and the existing evidence of differences in the quality of perinatal care received by women, patient‐level characteristics cannot fully explain the observed disparities in stillbirth without considering the context and content of healthcare received by these women. Women of low socioeconomic status are more likely to face obstacles such as being disadvantaged and vulnerable [28]; therefore, poverty could be the leading factor preventing equal access to maternity care. To address inequalities in maternity care, enhancement in living standards for disadvantaged women is required to provide access to education and increase employment opportunities [29]. Addressing the complex association between stillbirth and socioeconomic deprivation will depend upon understanding these underlying patient‐level factors influencing stillbirth [30]. Inequity in access to quality perinatal care due to mistrust of health services, language/communication difficulties, racial discrimination, poor nutrition, tobacco use, alcohol consumption and substance use could be central to these inequalities [31, 32, 33, 34, 35]. Several medical audits, including the Perinatal Confidential Enquiries, have been carried out in the UK, all of which highlight the importance of targeted perinatal care in reducing avoidable stillbirths [36, 37, 38, 39].

### 4.1 Clinical Implications

Ethnic minority mothers living in the most deprived areas had the highest risk of stillbirth. Though previous studies conducted in the UK [7, 40, 41] and other European countries [42, 43, 44] have identified ethnicity as an independent risk factor for stillbirth, this is the first study to report ethnic and socioeconomic disparities in stillbirth rates at the level of individual health trusts. Both ethnicity and socioeconomic deprivation predispose individuals to adverse pregnancy outcomes through complex mechanisms—biological, financial, social and cultural. However, it is apparent that some trusts with below average stillbirth rates for White women demonstrated above average stillbirth rates for Black and Asian women. This observed disparity in stillbirth rates within the same health trust suggests that inequalities in access and/or delivery of quality antenatal care may be an important target for intervention and improvement [45, 46].

### 4.2 Research and Health Policy Implications

The complex mechanisms responsible for the increase in stillbirth rates with ethnicity and socioeconomic deprivation do not necessarily mean that solutions should be complicated or impossible. Even though stillbirth rates are highest in Black women, 9% of pregnancy losses are formed by this ethnic group; the proportion of stillbirths was highest in White women (67.6%, *n* = 3000) and from the most deprived neighbourhoods (33.5%, *n* = 1495). This distribution of stillbirth suggests that targeting interventions based on ethnicity alone is unlikely to be effective in reducing stillbirth rates and could even perpetuate the flawed societal concept that ethnic and racial categories are biological determinants of health [47, 48]. The latter approach also runs the risk of stigmatisation of women on the basis of their ethnicity and also worsening the very health inequalities that need addressing [49]. A recent study reported a threefold reduction in perinatal death in Black and Asian women after early pregnancy risk personalised assessment using a model that included demographic, biophysical and biochemical characteristics [50, 51]. Targeting interventions based on risk prediction models that also include ethnicity and socioeconomic deprivation may represent the most effective approach towards stillbirth reduction. These findings should guide care and policymakers in addressing this challenge [29, 52].

### 4.3 Strengths and Limitations

Some trusts were smaller than others, and the number of mothers from specific ethnic and/or IMD groups also varied between trusts. However, this study is using data from all trusts in NHS England over several years and is therefore providing robust, representative evidence. This is not an aetiological study aiming to further our understanding of the causal factors of stillbirth but a report of the variability of stillbirth among mothers of different ethnicities and IMD groups. This study utilised the most reliable official routine maternity service data in England. Some studies have examined the geographical differences in stillbirths to quantify the neighbourhood effect on stillbirths [14, 53]. This study explores disparities across NHS Trusts and further considers inequality‐related factors. A limitation is that no causal relationship can be established as this is a descriptive study. Other inequality‐related factors, such as unemployment, cultural/religious beliefs, non‐English language and migration status, were not available in the data source used. Therefore, these factors could explain the inequalities observed. Ethnicity and IMD are likely to have a direct but also indirect relationship on stillbirth, and factors such as cultural beliefs, language barriers and access to care are likely to play an important role in the occurrence of this adverse outcome. However, this is a descriptive study that highlights inequalities that currently exist. Thus, disparities in stillbirth could be reduced by targeting populations that have higher than average rates of stillbirth as early as possible in the antenatal care pathway, as well as health trusts with demonstrable inequalities in care delivery. Some mitigation is provided using IMD metrics, which capture employment deprivation among other factors related to deprivation in the area where a woman lives. However, IMD is a broad measure and cannot give information specifically about the individual social class of women living in a particular area. Furthermore, we were unable to subdivide the presented ethnic groups and, therefore, could not examine internal variation within each ethnic group, thus potentially masking inequalities. In addition, the availability of individual‐level data for each woman, rather than the use of aggregated national data, allowed comparisons in stillbirth rates based upon maternal ethnicity and socioeconomic background. However, the number of livebirths and stillbirths was very small for some trusts, especially when examining the intersection between ethnicity and socioeconomic background; thus, there is an increased chance of false positives [54]. In this descriptive study, we solely focused on stillbirth rather than perinatal mortality, as neonatal deaths are typically less frequent and therefore prone to substantial variability. In addition, neonatal deaths are subjected to confounding by acute care in labour rather than antenatal care provision, which was the focus of this analysis.

## 5 Conclusion

Risk of stillbirth varied substantially by ethnic group and/or socioeconomic deprivation within individual health trusts. This is a descriptive study that highlights inequalities that currently exist, but as not all social and cultural confounders were available, inference cannot be established. This study emphasises the importance of considering factors that result in variability in delivery and/or access to healthcare at the level of the health trust. We also demonstrate that strategies to reduce stillbirth have to target both ethnic minority women and those who are socioeconomically deprived, if existing disparities are to be reduced. Findings from this study should guide care and policy stakeholders in prioritising interventions addressing the important public health challenge of reducing stillbirth.

## Author Contributions

All authors were involved in the conception, design and data collection of the study. G.K., E.L., and A.J. performed data analysis, and G.K., A.H., E.L., A.J. and B.T. drafted the first version of the manuscript. All authors critically reviewed the article and contributed to interpreting the results. The final version of the manuscript was submitted for publication after obtaining approval from all authors.

## Conflicts of Interest

The authors declare no conflicts of interest.

## Supporting information


Figure S1.

Figure S2.

Figure S3.

Figure S4.

Figure S5.

Figure S6.

Figure S7.

Figure S8.

Table S1.

## Data Availability

The data that support the findings of this study are available from the National Maternity and Perinatal Audit] but restrictions apply to the availability of these data, which were used under licence for the current study, and so are not publicly available. Data are, however, available from the authors upon reasonable request and with the permission of [National Maternity and Perinatal Audit].
