# Supplementary figures and images for: Disparities in Stillbirths in England: Analysis of A Population‐Based Study of 1.3 Million Births
Source: BJOG. 2025 May 16;132(8):1130–8. doi: 10.1111/1471-0528.18147 (PMC12137752; doi:10.1111/1471-0528.18147)

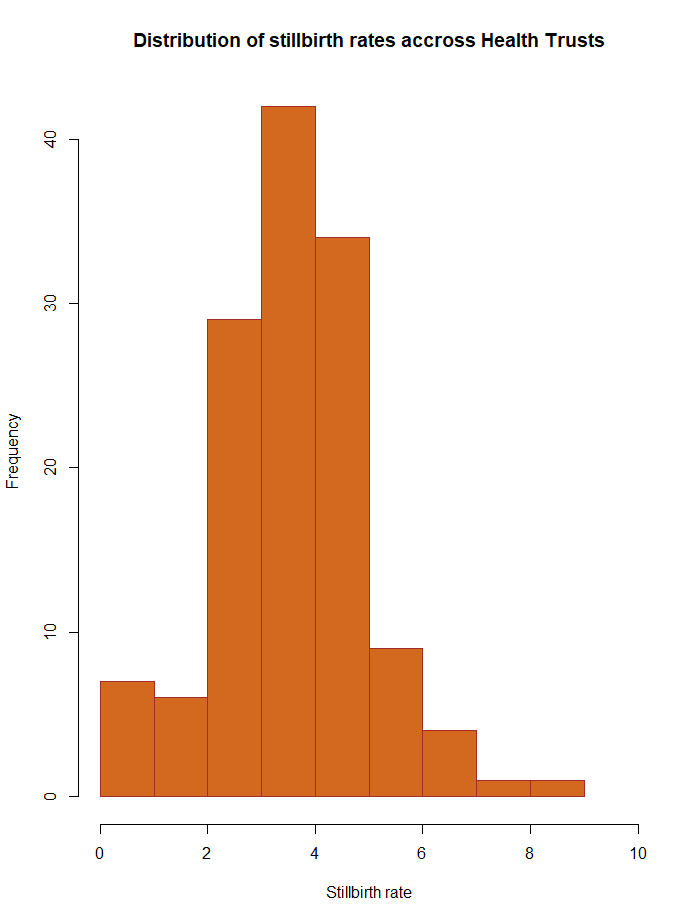

Supplement: Supplementary file 1 — Figure S1. [file BJO-132-1130-s009.png]

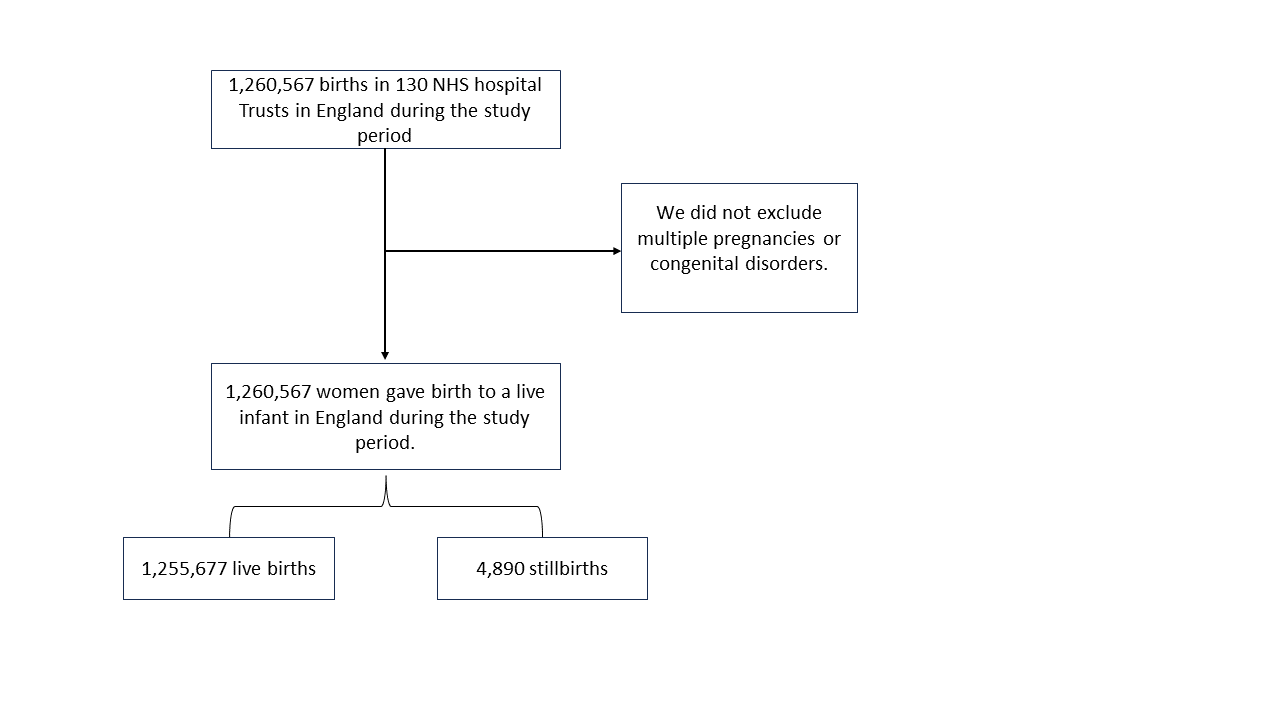

Supplement: Supplementary file 2 — Figure S2. [file BJO-132-1130-s002.png]

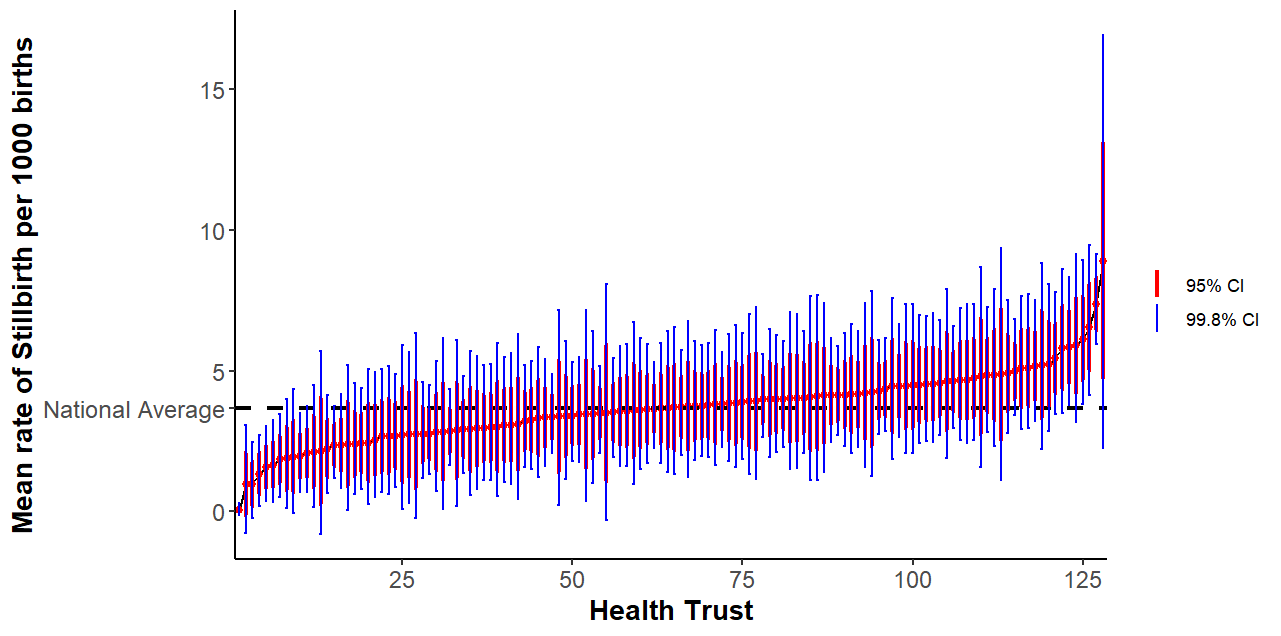

Supplement: Supplementary file 3 — Figure S3. [file BJO-132-1130-s008.tiff]

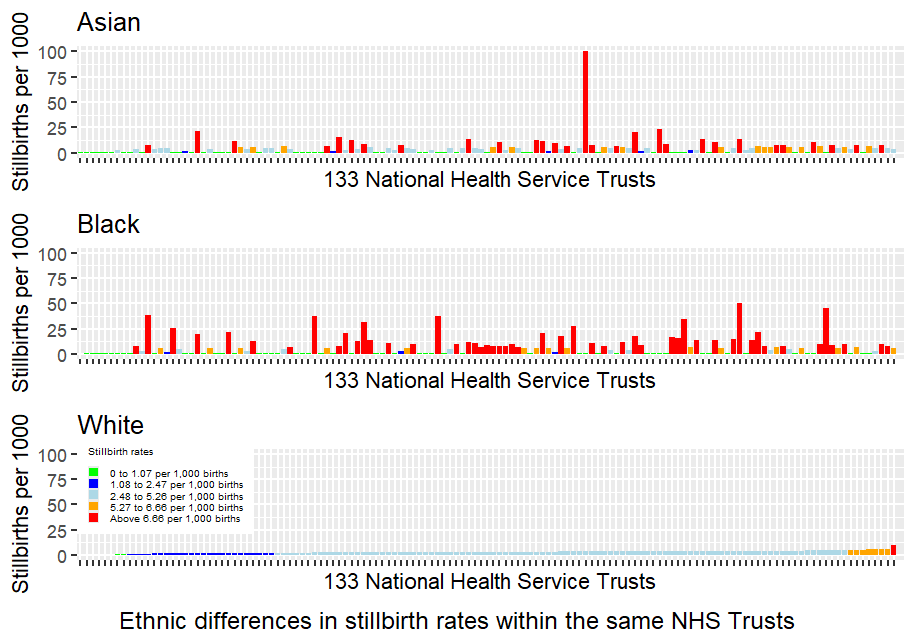

Supplement: Supplementary file 4 — Figure S4. [file BJO-132-1130-s003.tiff]

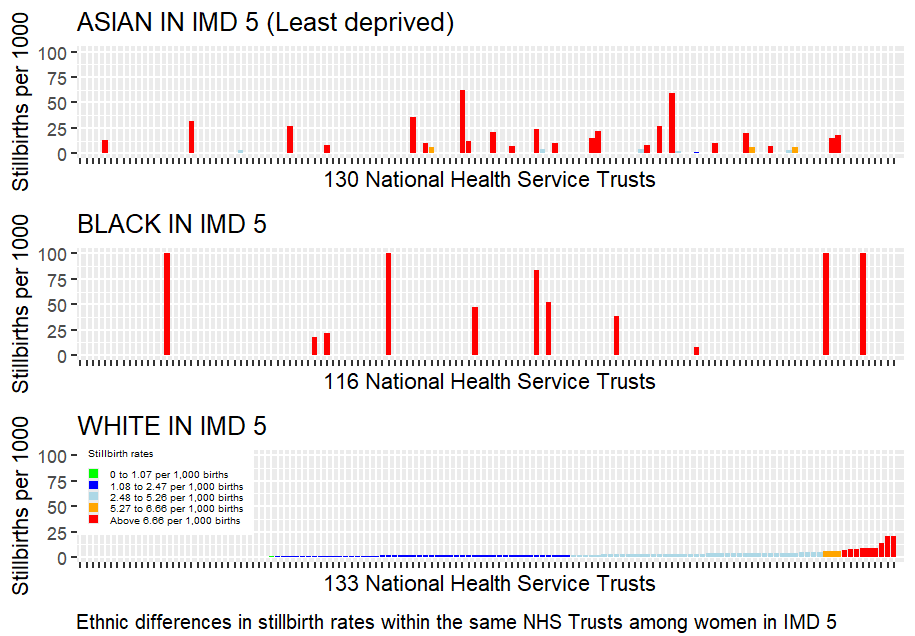

Supplement: Supplementary file 5 — Figure S5. [file BJO-132-1130-s005.tiff]

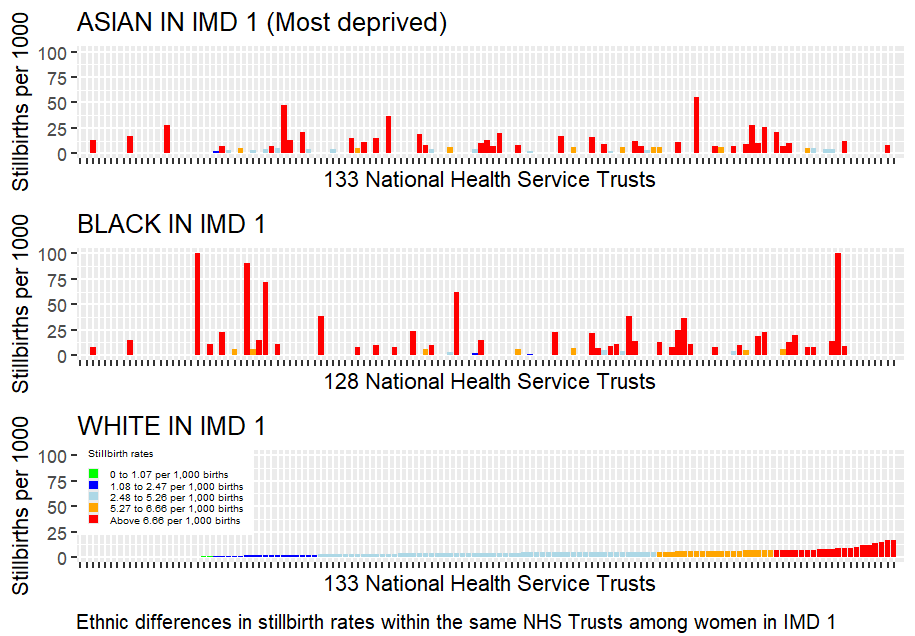

Supplement: Supplementary file 6 — Figure S6. [file BJO-132-1130-s006.tiff]

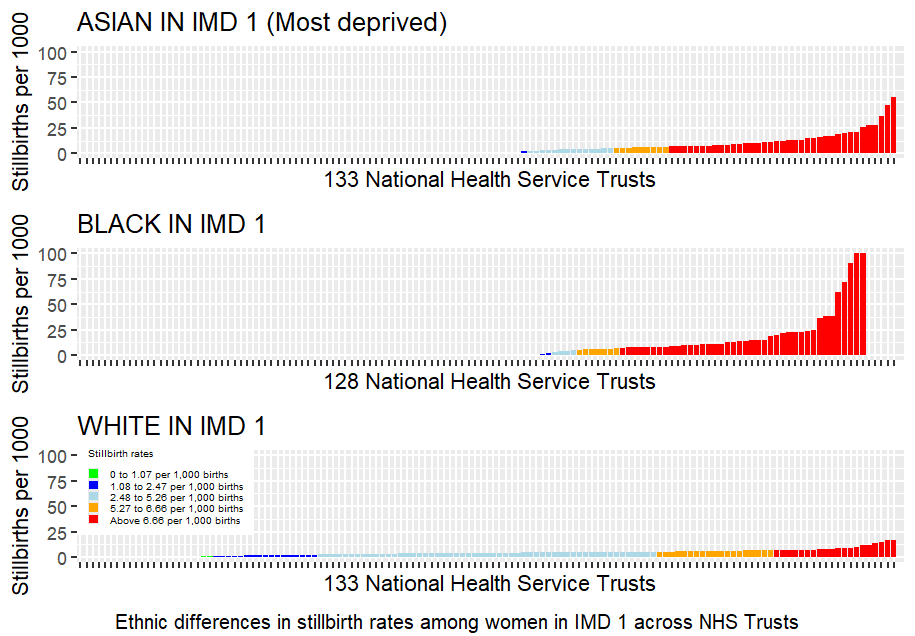

Supplement: Supplementary file 7 — Figure S7. [file BJO-132-1130-s007.tiff]

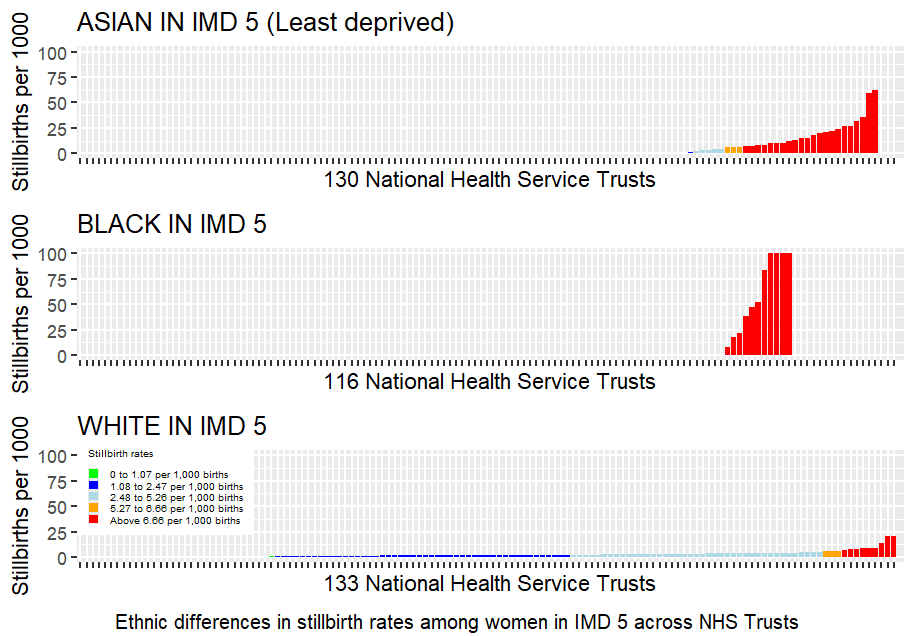

Supplement: Supplementary file 8 — Figure S8. [file BJO-132-1130-s004.tiff]
