# Supplementary material for: Disparities in Stillbirths in England: Analysis of A Population‐Based Study of 1.3 Million Births
Source: BJOG. 2025 May 16;132(8):1130–8. doi: 10.1111/1471-0528.18147 (PMC12137752; doi:10.1111/1471-0528.18147)
Supplement: Supplementary file 9 — Table S1. [file BJO-132-1130-s001.docx]

| **Supplementary Table 1: Ethnic and Socioeconomic Rates of Stillbirth across NHS Trusts*** | | | | | | | |
| --- | --- | --- | --- | --- | --- | --- | --- |
| **ETHNICITY** | | | | | | | |
| **Health Trust STILLBIRTH RATE** | **Number of Health Trusts** | **Total number of Stillbirths** | **ASIAN (n=741) ABSOLUTE RISK (95%CI)** | **BLACK (n=409)** | **MIXED RACE (n=97)** | **OTHER RACES (n=192)** | **WHITE (n=3000)** |
| Well below average ≥2SD  Below average <2SD  Average <1SD  Above average <2SD  Well above average ≥2SD | 7  19  95  10  2 | 7  322  3545  733  283 | 0.000 (0.000; 0.000)†  1.570 (1.567; 1.574)  4.140 (4.137; 4.142)  6.152 (6.150; 6.153)  12.305 (12.256; 12.353) | 0.000 (0.000; 0.000)†  1.640 (1.639; 1.642)  3.808 (3.804; 3.812)  6.241 (6.239; 6.243)  15.567 (15.538; 15.596) | 0.000 (0.000; 0.000)†  1.538 (1.538; 1.538)†  3.938 (3.934; 3.943)  6.290 (6.287; 6.292)  12.126 (12.109; 12.143) | 0.008 (0.008; 0.008)  1.645 (1.643; 1.647)  3.829 (3.826; 3.833)  6.137 (6.136; 1.139)  14.311 (14.274; 14.348) | 0.072 (0.071; 0.074)  1.679 (1.677; 1.681)  3.709 (3.708; 3.711)  6.039 (6.037; 6.041)  9.788 (9.788; 9.788)† |
| **SOCIOECONOMIC INDEX OF MULTIPLE DEPRIVATION (IMD)** | | | | | | | |
| **Health Trust STILLBIRTH RATE** | **Number of Health Trusts** | **Total number of Stillbirths** | **IMD1 (MOST DEPRIVED) (n=1495)** | **IMD2 (n=1063)** | **IMD3 (n=797)** | **IMD4 (n=595)** | **IMD5 (LEAST DEPRIVED) (n=508)** |
| Well below average ≥2SD  Below average <2SD  Average <1SD  Above average <2SD  Well above average ≥2SD | 7  19  95  10  2 | 7  322  3545  733  283 | 0.014 (0.014; 0.015)  1.843 (1.841; 1.845)  3.927 (3.925;3.930)  6.123 (6.121; 6.124)  10.014 (9.999; 10.029) | 0.131 (0.129; 0.132)  1.701 (1.700; 1.703)  3.830 (3.828; 3.832)  6.105 (6.103; 6.107)  8.592 (8.585; 8.599) | 0.151 (0.150; 0.153) 1.658 (1.657; 1.660)  3.842 (3.839; 3.843)  6.014 (6.013; 6.016)  8.313 (8.305; 8.322) | 0.096 (0.095; 0.097) 1.779 (1.777; 1.780)  3.601 (3.599; 3.602)  6.153 (6.150; 6.155)  9.746 (9.727; 9.765) | 0.000 (0.000; 0.000)† 1.703 (1.701; 1.704)  3.437 (3.435; 3.439)  6.119 (6.117; 6.122)  15.698 (15.637; 15.759) |
| * All rates are compared to the overall Stillbirth Rate of 3.4 stillbirths/1000 births measured across all NHS Trusts, using t-test. All presented rated were different from this national rate with p-value <0.00001  †Comparisons were not performed in those groups due to small group size  n = denotes the number of stillbirth per each Index of Multiple Deprivation or ethnic group | | | | | | | |
